# Acceptability of self-collected vaginal samples for human papillomavirus testing for primary cervical cancer screening: comparison of face-to-face and online recruitment modes

**DOI:** 10.1186/s12889-024-18551-5

**Published:** 2024-04-22

**Authors:** Siew-Fei Ngu, Lesley SK Lau, Ching Yin Chan, Hextan YS Ngan, Annie NY Cheung, Karen KL Chan

**Affiliations:** 1 Department of Obstetrics and Gynaecology, School of Clinical Medicine, The University of Hong Kong, Hong Kong, China; 2 LKS Faculty of Medicine, The University of Hong Kong, Hong Kong, China; 3 Department of Pathology, School of Clinical Medicine, The University of Hong Kong, Hong Kong, China

**Keywords:** Human papillomavirus, Cervical cancer screening, Self-sampling

## Abstract

**Background:**

This study aimed to assess the acceptability and attitudes of women towards human papillomavirus (HPV) self-sampling and compare the effectiveness of two delivery modes utilising face-to-face and online website for cervical cancer screening in Hong Kong.

**Methods:**

Women aged 30–65 years were invited to participate by distributing the study information pamphlets at the specialist clinics of a regional acute hospital. Those who were interested in participating were given the option to join directly face-to-face or through an online website. All participants provided informed consent and received self-sampling kits and acceptability questionnaires either immediately (face-to-face) or through the post after registering at the website (online). All participants were requested to collect their own vaginal samples using a swab which was then brushed on a DNA sample storage card and returned to the hospital either in person or by post. The self-collected samples were tested for high-risk HPV using the Sentis^™^ HPV assay, a validated isothermal nucleic acid amplification real-time fluorescent detection assay. The primary outcome was the uptake rate of HPV self-sampling.

**Results:**

Of the 1998 women recruited (1200 face-to-face, 798 online), 1377 returned their samples, giving an overall uptake rate of 68.9%. The uptake rate was significantly greater in the face-to-face mode than in the online mode (74.6% vs. 60.4%, *p* < 0.001). The median age of the participants was 49 years, 43.7% were never or under-screened, and 7.1% had high-risk HPV detected. Overall, 82.1% of the participants reported self-sampling convenient, and 79.3% were not embarrassed when collecting self-samples. However, only 49.8% were confident that they had collected the self-samples correctly. Most (91.1%) of the participants expressed willingness to perform self-sampling again, mostly because it was simple (79.2%) and quick (56.3%).

**Conclusions:**

HPV self-sampling can serve as an alternative primary screening method for cervical cancer in Hong Kong, especially for individuals who have not been adequately screened in the past. Both face-to-face and online website recruitment were associated with high acceptability, emphasising the potential benefits of utilising different platforms and strategies for reaching diverse populations.

## Background

Cervical cancer is the fourth most prevalent cancer in women worldwide, with approximately 604,000 new cases diagnosed and 342,000 fatalities reported in 2020 [1]. In Hong Kong, it ranks seventh among female cancers, accounting for 3.2% of new cases in 2020 and is the eighth leading cause of cancer-related mortalities [2]. Despite the introduction of population-based cervical screening in 2004 by the Department of Health, the incidence rate of cervical cancer has remained relatively stable with an age-standardised incidence rate of 8.5 per 100,000 standard population in 2020. Furthermore, a recent 2020/22 report of the Population Health Survey conducted in Hong Kong revealed that only 52.1% of females aged between 25 and 64 years had ever undergone cervical screening [3]. This screening coverage rate is significantly lower than the 70–80% achieved in other developed countries [4]. Multiple factors associated with non-attendance have been identified, including time constraints, inconvenience, embarrassment, pain, discomfort, cultural objections, transportation issues and cost [5]. This is concerning, as more than half of cervical cancer cases are diagnosed in women who have never or rarely undergone screening. Therefore, additional strategies have been investigated and implemented to improve cervical screening uptake and reduce cervical cancer incidence and mortality.

In recent years, many countries have adopted human papillomavirus (HPV)-based testing for cervical cancer screening, which has shown greater sensitivity than conventional cytology for detecting high-grade cervical intraepithelial lesions [6–9]. It also offers a longer screening interval and improves the negative predictive value of screening [10]. In Hong Kong, cervical cancer screening with either HPV testing or cytology or both (co-testing) is recommended in women aged 25 to 64 who ever had sexual experience. Recently, in April 2023, the Department of Health in Hong Kong has implemented primary HPV testing in their cervical cancer screening programme, particularly for women aged 30 to 64 [11]. For women aged 25 to 29, cervical cytology is recommended due to their higher prevalence of HPV infection which are mostly transient. Nonetheless, primary HPV screening can be considered in this group of women if they had previously received HPV vaccination [12]. With the introduction of HPV-based screening, self-sampling has emerged as an alternative strategy, allowing women to collect vaginal samples for HPV testing, thereby enhancing screening coverage [13]. Self-sampling has the potential to mitigate some of the barriers faced by non-attenders, as it can be conducted in the privacy of a woman’s own home without attending healthcare settings [14, 15]. Furthermore, a recent meta-analysis revealed that the sensitivity and specificity of HPV testing on self-samples were comparable to those of clinician-collected samples, especially when validated PCR-based HPV tests were used [16, 17].

In an attempt to feasibly implement self-sampled HPV tests in a local screening programme, this study aimed to assess the acceptability and attitudes of women towards HPV self-sampling and compare the effectiveness of two delivery modes utilising face-to-face and online website for cervical cancer screening in Hong Kong.

## Methods

### Study design

This acceptability study was approved by the local institution’s ethics review board (The University of Hong Kong and Hospital Authority Hong Kong West Cluster Institutional Review Board) and conducted in accordance with the ethical principles of the Declaration of Helsinki. The primary outcome was the uptake rate of HPV self-sampling, calculated by the number of samples returned/the number of self-samples kits distributed. The secondary outcomes were the effectiveness of two delivery modes including face-to-face and online website, the prevalence of HPV infections in the self-collected samples, and the attitudes of women towards HPV self-sampling.

### Study population

We aimed to recruit around 2000 women from the general public who came to the specialist clinics at Queen Mary Hospital (a regional acute hospital that also serves as a territory-wide tertiary and quaternary referral centre for many complex and advanced services and teaching hospital) as patients, accompanying persons or hospital staff. Women were recruited over a period of around 3 months between 13th October 2022 and 17th January 2023. Women aged 30–65 years with a history of sexual activity were eligible for the study regardless of their screening history. Individuals who had a history of hysterectomy, were currently pregnant or menstruating, had symptoms of cervical cancer or were receiving treatment for cervical dysplasia or cancer were excluded.

### Study intervention

**Fig. 1:**
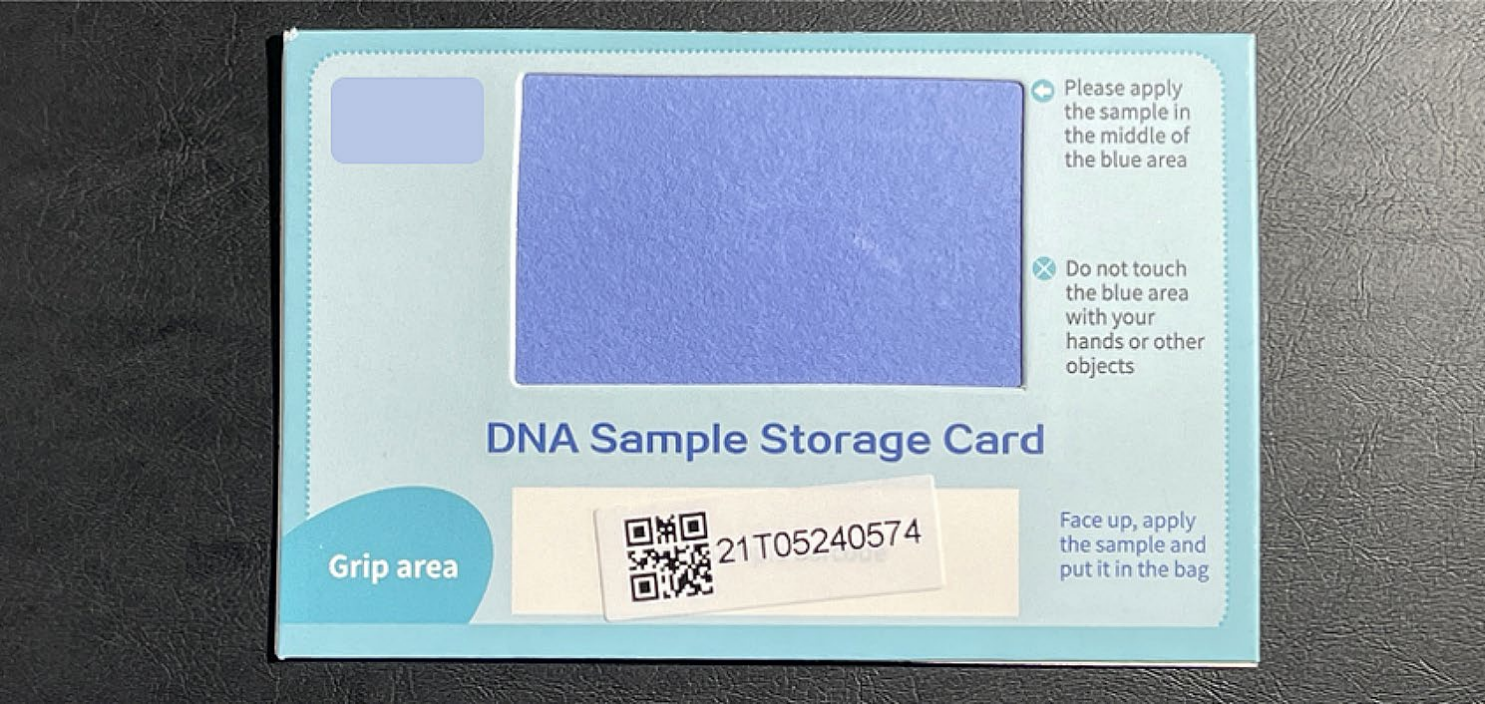
A DNA sample storage card

Women were given information regarding the study using an information pamphlet (available in English and Chinese), which contained a QR code to an online website. Eligible women were recruited by the research assistants who were experienced in clinical trials on gynaecological malignancy and have basic knowledge of cervical cancer screening and HPV self-sampling. Those who were interested in participating were given the option to join directly face-to-face or through the online website. All participants were given a self-sampling toolkit free of charge which included a set of diagrammed instructions for self-sampling, a sealed long sterile swabs (Shenzhen Medico Technology Co. Ltd, China), and a DNA Sample Storage Card (solid transport media) (BGI Biotechnology (Wuhan) Co. Ltd., China), which the participants used to collect specimens from the swabs (Fig. 1). The participants were instructed to insert the swab into the upper vagina, rotate three to five times, and then remove the swab from the vagina. The swab was then brushed back and forth over the blue sample collection patch on the DNA Sample Storage card and left to dry in a safe place. The dried DNA sample storage card was then stored in a Ziploc bag provided. Participants were also requested to complete an acceptability questionnaire that included sociodemographic data, cervical screening history and attitudes towards their experience with self-sampling. Acceptability was assessed using a five-point Likert scale that included a range of subjective qualities such as convenience, embarrassment, confidence, discomfort, and overall experience.

#### Face-to-face mode

Participants who agreed to participate directly face-to-face completed a written consent form and watched an instructional video on how to collect the self-samples. The participants were then given a self-sampling toolkit and questionnaire by the research assistants, from which they could choose to perform self-sampling on their own privacy in the hospital or at home. The participants were requested to return the self-collected specimen and completed questionnaire to a designated place at Queen Mary Hospital either in person or by post. All self-collected specimens underwent HPV testing.

#### Online website mode

Eligible women were requested to register and provide their informed consent at a mobile-friendly online website. Apart from the information of the study, the website also contained general information about cervical cancer screening. Participants subsequently received the self-sampling kits and questionnaires through the post. The participants were instructed to watch an instructional video on how to collect self-samples, which were available on the website. Thereafter, they could perform self-sampling at their own privacy at home, and then return the self-collected specimen and completed questionnaire to a designated place at Queen Mary Hospital by mail. All self-collected specimens underwent HPV testing.

#### HPV testing

All self-collected vaginal specimens were tested for 14 high-risk HPV subtypes (specific genotyping of HPV 16 and 18, and pooled detection of HPV 31, 33, 35, 39, 45, 51, 52, 56, 58, 59, 66 and 68) using Sentis™ HPV assay. It is an isothermal nucleic acid amplification real-time fluorescent detection assay utilising the AmpFire HPV assay (Atila Biosystems, USA). Specific primers and probes were used to amplify regions of viral genomic DNA including E7/E8 regions. The assay was performed according to the manufacturer’s instructions by the BGI Health (HK) Company Limited in Hong Kong. The validation on the accuracy and reproducibility of this HPV assay for cervical cancer screening has been reported previously by our group [18] and other researchers [19].

#### Follow-up procedure

All participants were informed of their HPV test results electronically via email or short messaging services. As self-sampling is not yet a recommended method for cervical cancer screening in Hong Kong, women who tested positive for high-risk HPV were informed that the results may indicate an increased chance of developing cervical abnormalities and were advised to have a standard cervical sampling by a health care provider. Women without high-risk HPV detected were advised to have regular cervical smear. Participants who did not return the self-collected samples were sent a reminder 6 to 8 weeks after giving out the toolkits, and again 2 weeks before the closure of the study.

#### Statistical analysis

Participants with incomplete data (inadequate specimen, failure of the HPV test, incomplete questionnaire) were excluded from the analysis. All the data were collected and analysed using Statistical Package for the Social Sciences version 26.0 (SPSS Inc., Chicago, Il, USA). The differences in proportions amongst the different groups were calculated using chi-square test or Mann-Whitney U tests with significance level of 0.05.

## Results

### Demographics and response rate

The demographic data of the participants are shown in Table 1. A total of 1998 eligible women were recruited between October 2022 and January 2023; 1200 were recruited face-to-face, and 798 were recruited via the online website. Of these, 1377 women returned self-collected samples, giving an overall uptake rate of 68.9% (Table 2). Based on the mode of recruitment, the uptake rate was 74.6% and 60.4% in the face-to-face and online website, respectively. Seventeen participants who returned the samples had invalid results and declined resampling. The median age of the participants was 49 years among all participants, 46 years among women recruited online and 50 years among women recruited face-to-face. The proportion of women in the younger age group was significantly greater among those recruited online than among those recruited face-to-face (24.5% vs. 16.7% in 30–40 age group; 49.0% vs. 32.2% in 41–50 age group, *p* < 0.001). Most (97.8%) of the participants were Chinese, and 69.0% were multiparous. Regarding the screening history, 545 (43.7%) women were never or under-screened (no screening for more than three years) and 603 (48.3%) had regular screening (had screening within three years). There was no significant difference in the proportion of never or under-screened women between those recruited via the face-to-face mode and those recruited via the online mode.

**Table 1:** Demographic data of the participants by delivery mode

Demographics	Face-to-face recruitment, *N* = 819 n (%)	Online website recruitment, *N* = 429 n (%)	Total, *N* = 1248 n (%)	*P* value*
**Median age**, years (range)	50 (30–65)	46 (30–65)	49 (30–65)	< 0.001
**Age group**				
30–40	137 (16.7)	105 (24.5)	242 (19.4)	< 0.001
41–50	264 (32.2)	210 (49.0)	474 (38.0)	
51–65	392 (47.9)	113 (26.3)	505 (40.5)	
Not specified	26 (3.2)	1 (0.2)	27 (2.2)	
**Ethnicity**				
Chinese	797 (97.3)	423 (98.6)	1220 (97.8)	0.209
Non-Chinese	3 (0.4)	6 (1.4)	9 (0.7)	
Unknown	19 (2.3)	0 (0.0)	19 (1.5)	
**Monthly income**				
< $10,000	48 (5.9)	16 (3.7)	65 (5.2)	0.001
$10,000 - $19,999	191 (23.2)	65(15.0)	256 (20.4)	
$20,000 - $29,999	139 (16.9)	74 (17.1)	213 (17.0)	
$30,000 - $39,999	86 (10.4)	50 (11.6)	136 (10.8)	
≥ $40,000	183 (22.2)	128 (29.6)	311 (24.8)	
Missing	176 (21.4)	99 (22.9)	275 (21.9)	
**Education**				
Primary or below	63 (7.7)	9 (2.1)	72 (5.8)	< 0.001
Secondary	473 (57.8)	225 (52.4)	698 (55.9)	
Tertiary	168 (20.5)	128 (29.8)	296 (23.7)	
Postgraduate or above	57 (7.0)	32 (7.5)	89 (7.1)	
Missing	58 (7.1)	35 (8.2)	93 (7.5)	
**Parity**				
0	165 (20.1)	123 (28.7)	288 (23.1)	< 0.001
≥ 1	556 (67.9)	305 (71.1)	861 (69.0)	
Missing	98 (12.0)	1 (0.2)	99 (7.9)	
**Screening history**				
Never screened	116 (14.2)	47 (11.0)	163 (13.1)	< 0.001
Under screened	233 (28.4)	149 (34.7)	382 (30.6)	
Regular screening	389 (47.5)	214 (49.9)	603 (48.3)	
Missing	81 (9.9)	19 (4.4)	100 (8.0)	

*P value for comparison between face-to-face recruitment and online website recruitment

**Table 2:** Recruitment and uptake rate of HPV self-sampling

Recruitment	Face-to-face, n (%)	Online website, n (%)	Total, n (%)	*P* value
No. of participants	1200	798	1998	
Samples returned	895 (74.6)	482 (60.4)	1377 (68.9)	< 0.001
Samples with results	882 (73.5)	478 (59.9)	1360 (68.1)	< 0.001
Questionnaire completed	827 (92.1)	432 (89.6)	1256 (91.2)	0.154

### Prevalence of HPV infections

High-risk HPV was detected in 96 (7.1%) samples, 9 (9.4%) were positive for HPV 16 or 18, and 92 (95.8%) were positive for other pooled types of high-risk HPV (Table 3). Four women had coinfection with HPV 16 and other pooled HPV types, and one woman had coinfection with HPV 18 and other pooled HPV types. The prevalence of HPV was not significantly different across the different age categories, where 17 (7.1%), 24 (5.1%) and 37 (7.4%) women tested positive in the 30–40, 41–50 and 51–65 age group, respectively.

**Table 3:** Prevalence of high-risk HPV infections in self-collected samples

	Face-to-face, n (%)	Online website, n (%)	Total, n (%)
HPV positive	70 (7.9)*	26 (5.4)	96 (7.1)
HPV 16	6 (8.6)	0 (0.0)	6 (6.3)
HPV 18	2 (2.9)	1 (3.8)	3 (3.1)
Other high-risk HPV^#^	67 (95.7)	25 (96.2)	92 (95.8)
HPV negative	812 (92.1)	452 (94.6)	1264 (92.9)

*4 participants had coinfection with HPV16 and other high-risk HPV, 1 had coinfection with HPV18 and other high-risk HPV

^#^Other high-risk HPV includes HPV 31, 33, 35, 39, 45, 51, 52, 56, 58, 59, 66 and 68

### Acceptability

Out of the 1377 women who returned self-collected samples, 1256 (91.2%) completed the acceptability questionnaire. Among these respondents, 82.1% reported that self-sampling was convenient or very convenient, 79.3% expressed that they were not embarrassed or not embarrassed at all when performing self-sampling, and 61.6% found self-sampling easy or very easy to perform (Table 4). Only 49.8% of women expressed that they were confident or very confident in performing self-sampling accurately. There were significantly greater proportions of women who reported self-sampling easy or very easy to perform (65.5% vs. 59.6%, *p* = 0.047), reported no discomfort or no discomfort at all (58.0% vs. 48.6%, *p* = 0.002) and had overall good or very good experience with self-sampling (54.6% vs. 45.5%, *p* = 0.002) among women who were recruited online than among those recruited face-to-face.

**Table 4:** Acceptability of HPV self-sampling

Attributes	Face-to-face, *N* = 819 n (%)	Online website, *N* = 429 n (%)	Total, *N* = 1248 n (%)	*P* value
Convenient or very convenient	659 (80.5)	365 (85.1)	1024 (82.1)	0.052
Not embarrassed or not embarrassed at all	637 (77.8)	353 (82.3)	990 (79.3)	0.073
Easy or very easy	488 (59.6)	281 (65.5)	769 (61.6)	0.048
No discomfort or no discomfort at all	398 (48.6)	249 (58.0)	647 (51.8)	0.002
Confident or very confident	420 (51.3)	202 (47.1)	622 (49.8)	0.178
Overall good or very good experience	375 (45.5)	236 (54.6)	611 (48.6)	0.002

When analysing the acceptability across different age groups, there were significantly greater proportions of women in the younger age group of 30–40 who reported self-sampling easy or very easy to do (66.1% vs. 54.5%, *p* = 0.003), were confident or very confident that they had collected their self-samples correctly (53.7% vs. 45.5%, *p* = 0.044), reported no discomfort or no discomfort at all while collecting their self-samples (55.4% vs. 43.6%, *p* = 0.003), and had overall good or very good experience with self-sampling (50.4% vs. 41.0%, *p* = 0.019) compared to the women in the older age group of 51–65 (Table 5). In contrast, among women aged 41–50 years, a significantly greater proportion were not embarrassed or not embarrassed at all to collect self-samples (83.1% vs. 76.4%, *p* = 0.041) than was found in the younger group of 30–40 years.

**Table 5:** Acceptability of HPV self-sampling in different age groups

Attributes	Age 30–40 *N* = 242	Age 41–50 *N* = 474		Age 51–65 *N* = 505	
	n (%)	n (%)	P value	n (%)	P value
Easy or very easy	160 (66.1)	319 (67.3)	0.815	275 (54.5)	0.003
Convenient or very convenient	203 (83.9)	405 (85.4)	0.659	397 (78.6)	0.110
Not embarrassed or not embarrassed at all	185 (76.4)	394 (83.1)	0.041	388 (76.8)	0.981
Confident or very confident	130 (53.7)	252 (53.2)	0.951	230 (45.5)	0.044
No discomfort or no discomfort at all	134 (55.4)	279 (58.9)	0.416	220 (43.6)	0.003
Overall good or very good experience	122 (50.4)	266 (56.1)	0.171	207 (41.0)	0.019

In terms of parity, a significantly greater proportion of multiparous women reported self-sampling as easy or very easy to perform (64.5% vs. 55.2%, *p* = 0.006), were confident or very confident that they had collected their self-samples correctly (52.0% vs. 43.8%, *p* = 0.018) and had overall good or very good experience with self-sampling (50.6% vs. 43.4%, *p* = 0.039) compared to the proportions of primiparous women.

Concerning the participants’ most preferred screening methods, 35.7% expressed no preference, 34.0% preferred self-collected vaginal swabs for HPV testing (self-sampling), 21.6% preferred standard physician-collected cervical smears, and 8.6% preferred physician-collected vaginal swabs for HPV testing (Table 6). Across the different age groups, there were greater preferences for self-sampling than for standard physician-collected cervical smears (34.7% vs. 16.9% in 30–40 years; 33.2% vs. 22.3% in 41–50 years and 34.2% vs. 23.4% in 51–65 years). Among women who were never or under-screened, a greater proportion preferred self-sampling, while among those who had regular screening, a greater proportion had no preference for the screening method (results not shown). Compared to those recruited through face-to-face interactions, women who were recruited online had a greater preference for self-sampling, particularly among women who were never or under-screened (51.1% vs. 44.3% in never screened; 46.3% vs. 35.7% in under-screened).

Overall, the majority (91.1%) of women expressed their willingness to undergo self-sampling again, mainly because they found self-sampling simple (79.2%) and quick (56.3%) (Table 6). Regarding participants’ willingness to undergo self-sampling based on their recruitment method, a significantly greater proportion of women who were recruited online expressed their willingness to self-sample again, than did those recruited face-to-face (96.5% vs. 88.3%, p = < 0.001). However, 8.1% of participants were not willing to self-sample again, citing reasons for lack of confidence in taking self-samples accurately (65.3%) or a preference for healthcare providers to take the samples (38.6%).

**Table 6:** The most preferred method for cervical cancer screening and willingness to undergo self-sampling again

	Face-to-face, n (%)	Online website, n (%)	Total, n (%)	P value
**Most preferred screening method**				
No preference	281 (35.1)	158 (36.8)	439 (35.7)	0.081
Self-collected vaginal swab for HPV testing	265 (33.1)	153 (35.7)	418 (34.0)	
Physician-collected cervical smear	173 (21.6)	93 (21.7)	266 (21.6)	
Physician-collected vaginal swab for HPV testing	81 (10.1)	25 (5.8)	106 (8.6)	
**Would you be willing to do the HPV self-sampling test again?**				
***Yes***	**723 (88.3)**	**414 (96.5)**	**1137 (91.1)**	**< 0.001**
*Reason*				
The test is simple to do	567 (78.4)	334 (80.7%)	901 (79.2)	< 0.001
The test is quick	419 (58.0)	221 (53.4%)	640 (56.3)	0.152
I feel more comfortable taking own sample	340 (47.0)	194 (46.9%)	534 (47.0)	1.000
I feel less embarrassed taking own sample	343 (47.4)	169 (40.8%)	512 (45.0)	0.036
Taking sample with swab was not painful	267 (36.9)	139 (33.6%)	406 (35.7)	0.284
I am confident that I can take own sample accurately	245 (33.9)	113 (27.3%)	358 (31.5)	0.025
***No***	**86 (10.5)**	**15 (3.5)**	**101 (8.1)**	
*Reason*				
I am not confident to take own sample accurately	58 (67.4)	8 (53.3)	66 (65.3)	0.444
I prefer a healthcare professional to collect the sample	33 (38.4)	6 (40.0)	39 (38.6)	1.000
I am afraid I might hurt myself	25 (29.1)	6 (40.0)	31 (30.7)	0.587
Taking own sample with the swab was painful	19 (22.1)	3 (20.0)	22 (21.8)	1.000
The test is not easy	16 (18.6)	5 (33.3)	21 (20.8)	0.341
I am not comfortable taking own sample	9 (10.5)	1 (6.7)	10 (9.9)	1.000

## Discussion

This study included 1998 women with HPV self-sampling and demonstrated an overall uptake rate of 69%, with a higher uptake rate among participants recruited from face-to-face interactions (75%) than among those recruited from online website (60%). There was a significantly greater proportion of women in the younger age group among those recruited online than among those recruited face-to-face. Never-screened and under-screened women accounted for 44% of the total participants, and greater proportion of these women preferred self-sampling compared to women who had regular screening. Around 80% the participants found self-sampling convenient and were not embarrassed when collecting self-samples, although only 50% were confident that they had collected the self-samples correctly. Additionally, majority (91%) of participants were willing to perform HPV self-sampling again, and it was the most preferred screening method in around one-third of women.

The uptake rate of 69% in this study is comparable to that of a previous local study in which an overall uptake rate of 62% was reported among under-screened women recruited through social media, school outreach programmes and underserved outreach through nongovernmental organisations [20]. These uptake rates are considerably greater than those reported in a meta-analysis in which the pooled participation rates ranged between 8% and 20% when the self-sampling kit was mailed to women’s homes, when the women had to request a self-sampling kit, or when they were invited through community campaigns [17]. However, compared to pooled participation rate of 95%, the uptake rate in our study was lower when the community health workers delivered self-sampling kits directly to women’s homes or workplaces. The uptake rate among participants recruited via face-to-face mode in our study where the kits were given directly to the women was relatively lower than the pooled participation rate of 95%, probably because they have the option to collect the samples in their own privacy at home instead of returning the samples immediately. Furthermore, as the self-sampling kits were distributed free of charge, some women may have joined the study to obtain a free sample out of curiosity without the intention to perform the test.

Owing to the relatively low uptake (52%) of cervical cancer screening in Hong Kong [3], identifying and reaching out those never or under-screened women who are at higher risk for cervical cancer are paramount to achieve the goal of eliminating cervical cancer by 2030 according to the World Health Organisation [21]. HPV self-sampling has been successfully implemented in both high- and low-income countries in recent decades and has been shown to be a cost-effective screening strategy [14, 22]. HPV self-sampling offers numerous advantages, including convenience, less embarrassment, reduced costs, flexibility in performing the test at home, avoidance of the need for pelvic examination which can be uncomfortable, and overcoming some social and cultural barriers [15, 23].

The prevalence of high-risk HPV in the current study was 7.1%. According to a recent local study on cervical cancer screening involving 15,955 women aged 30–60 years, the prevalence of high-risk HPV in cervical samples was 8.7% [6], which is comparable to that in the current study, probably reflecting the similar study population between the two studies. In another local study including 321 under-screened women aged 30–65 years, the prevalence of high-risk HPV in clinician-collected cervical samples and self-collected vaginal samples was 12.9% and 10.9%, respectively [20], which is greater than that in the current study, as it included only under-screened women. The self-collected vaginal samples and HPV assay in the present study had been compared to the “gold standard” of clinician-collected cervical samples and an HPV assay (BD Onclarity™ HPV Assay) that was approved by the United States Food and Drug Administration (FDA), with substantial agreement (concordance of 84%, kappa of 0.64) reported. A recent large, randomised study comparing self-sampling and clinician-collected cervical samples conducted in the Netherlands supported the use of HPV self-sampling as a primary screening method in routine cervical cancer screening with similar accuracy for detecting high-grade cervical intraepithelial lesions [24].

Notably, women recruited through online website were significantly younger than women who joined directly face-to-face. In addition, women in the younger age group contributed to greater acceptability and preference for self-sampling, particularly in terms of ease, discomfort, confidence, and overall experience. Nonetheless, in our study, more than 70% of the participants in the older age group of 51–65 found self-sampling convenient and not embarrassing. A recent Swedish study on the perception of HPV self-sampling among older women (aged 64 years and older) reported confidence in self-sampling, and that self-sampling was comfortable, convenient as well as time and money saving [25]. In this study, 91% of participants were willing to perform HPV self-sampling again, and HPV self-sampling was the most preferred screening method in around one-third of women, which is consistent with the results of previous local studies [20]. Moreover, nearly half of the participants had never undergone cervical screening or were under-screened. Based on these results, HPV self-sampling has the potential to increase screening coverage and could be considered an option for cervical cancer screening in Hong Kong. Nonetheless, it is critical to involve key stakeholders including healthcare providers, policymakers, and target populations in developing acceptable delivery strategies for implementing HPV self-sampling in screening programmes to ensure demand and facilitate uptake from eligible women. The online website mode where women can request a self-sampling kit is a promising strategy, especially for younger women, as demonstrated in this study, and has the benefit of requiring less resources such as manpower and facilities, than the face-to-face mode.

Overall, our results highlighted the favourable acceptability and attitudes toward HPV self-sampling among the study participants. With the recent COVID-19 pandemic where self-collected nasal swabs for virus testing have become commonplace in the daily life of many people around the world, self-collected vaginal swabs may also become more acceptable. The majority of participants found self-sampling convenient and not embarrassing. However, there is a need to address women’s level of confidence in performing self-sampling and potentially develop strategies to increase comfort and ease during self-sampling procedures, particularly among women in older age groups. These findings may help to inform interventions and educational efforts aimed at improving the acceptability and promoting the wider adoption of HPV self-sampling for cervical cancer screening.

Nevertheless, it is important to acknowledge some limitations of this study. First, the study design relied on self-reported data, which may be subject to recall bias or social desirability bias. Second, the sample size and recruitment methods may have introduced selection bias. Additionally, this study only focused on the acceptability and attitudes toward self-sampling and did not explore the clinical outcomes or cost-effectiveness of this approach. Future research should explore the cost-effectiveness of self-sampling, considering the preferences and needs of different population groups. Tailoring interventions to address age-related differences and improving self-sampling confidence could further enhance acceptability.

## ‌Conclusions

In conclusion, the findings of this study indicate that HPV self-sampling can serve as an alternative primary screening method for cervical cancer in Hong Kong, especially for individuals who have not been adequately screened in the past. Both face-to-face and online website recruitment were associated with high acceptability, emphasising the potential benefits of utilising different platforms and strategies for reaching diverse populations.

## Data Availability

All data generated or analysed during this study are included in this published article.
